# Intra-osteal fixation of comminuted coronoid process fracture with mini plate for the treatment of complex elbow fracture

**DOI:** 10.1093/jscr/rjae571

**Published:** 2024-09-05

**Authors:** Yuxuan Wu, Jianlan Wang, Xiaodong Lian, Jiang Mu, Ming Lu, Shuang Zhang

**Affiliations:** Department of Orthopedics, Dalian Municipal Central Hospital Affiliated of Dalian University of Technology, Dalian, Liaoning 116024, China; Department of Traditional Chinese Medicine, Dalian Municipal Central Hospital Affiliated of Dalian University of Technology, Dalian, Liaoning 116024, China; Department of Orthopedics, Dalian Municipal Central Hospital Affiliated of Dalian University of Technology, Dalian, Liaoning 116024, China; Department of Orthopedics, Dalian Municipal Central Hospital Affiliated of Dalian University of Technology, Dalian, Liaoning 116024, China; Department of Orthopedics, Dalian Municipal Central Hospital Affiliated of Dalian University of Technology, Dalian, Liaoning 116024, China; Department of Orthopedics, Dalian Municipal Central Hospital Affiliated of Dalian University of Technology, Dalian, Liaoning 116024, China

**Keywords:** coronoid process fracture, intra-osteal fixation, complex elbow fracture

## Abstract

Complex elbow fractures featuring a comminuted coronoid process are infrequent and pose considerable treatment challenges. The optimal strategy for maximizing recovery of elbow function through osteosynthesis remains a subject of ongoing debate among surgeons. We applied the principle of internal fixation by implementing intra-osteal fixation with a mini plate, which facilitated the successful restoration of exceptional elbow function in the patient. This approach adeptly managed the complexity of the coronoid process fracture, encompassing its fragmentation and associated injuries, thereby demonstrating its feasibility and efficacy in achieving favorable clinical outcomes. This article investigates the viability of this surgical technique for managing such complex fractures.

## Introduction

The coronoid process is a critical stabilizing structure situated at the anterior aspect of the elbow joint. The incidence of coronoid process fractures ranges from 2 to 15%, frequently occurring in conjunction with fractures of the olecranon or radial head, ligamentous damage, and elbow dislocation, which collectively contribute to significant joint instability [1]. Inadequate management of complex coronoid process fractures may result in chronic elbow instability, diminished range of motion, and prolonged functional impairment. Achieving optimal fracture repair while preserving elbow mobility is particularly challenging, due to the presence of intra-articular fractures and the intricate anatomy surrounding the joint. Recently, we employed mini plate intra-osteal fixation to address a complex elbow fracture compounded by a comminuted coronoid process fracture, resulting in significant improvement in elbow function for the patient. This paper provides a comprehensive overview of the treatment approach and evaluates the feasibility of intra-osteal fixation for coronoid process fractures.

## Case report

**Figure 1:**
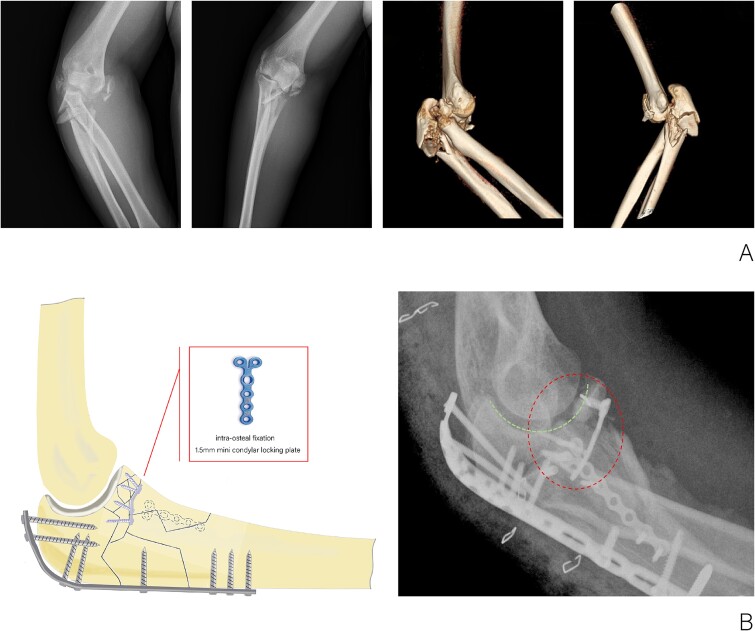
(A) X-ray and CT three-dimensional reconstruction examination of elbow fracture. (B) Diagram of intra-osteal fixation of comminuted coronoid process fracture with mini plate and postoperative X-ray examination of the elbow.

The patient, a 36-year-old male, sustained a direct trauma to the elbow joint resulting from a forceful fall and impact with the ground. The diagnosis revealed a right Monteggia fracture, accompanied by posterior elbow dislocation and ulnar nerve injury. Examination revealed no external wounds on the elbow joint. Furthermore, preoperative assessment confirmed that the blood supply, ulnar nerve, radial nerve, and central nerves of the affected limb remained intact. A plaster was used to fix the elbow in the flexor position temporarily. Three-dimensional CT reconstruction clearly indicated an O’Discoll III fracture of the coronoid process, which was fragmented into three discrete bone fragments. Additionally, there was evidence of a comminuted fracture of the olecranon as well as a fracture of the radial head (Fig. 1A).

The surgical procedure was conducted under a combination of brachial plexus block anesthesia and general anesthesia, with the application of an upper arm tourniquet. A posterior cubital incision was made to access the fracture and safeguard the ulnar nerve. The primary fracture fragments of the coronoid process measured 8 × 9, 15 × 9, and 10 × 9 mm^2^, respectively. A three-hole, 1.5-mm mini condylar locking plate and a 1.0-mm Kirschner wire were utilized to stabilize the coronoid process in conjunction with the olecranon intra-osteally, aiming to reconstruct the joint surface. A proximal dorsal ulna locking compression plate was employed to secure the olecranon to the distal ulna from the dorsal aspect. Additionally, a large medial fracture block of the proximal ulna was stabilized using a six-hole, 1.5-mm mini condylar locking plate. Subsequently, the radial head was exposed and its fracture was stabilized with a 2.0-mm cannulated screw. The elbow drawer test and stress test results were negative, suggesting the absence of instability related to injuries of the medial and lateral collateral ligaments and the anterior capsule. Consequently, exploration of the collateral ligaments was deemed unnecessary and external fixation of the elbow was not performed. The duration of the surgical procedure was approximately 100 min.

**Figure 2:**
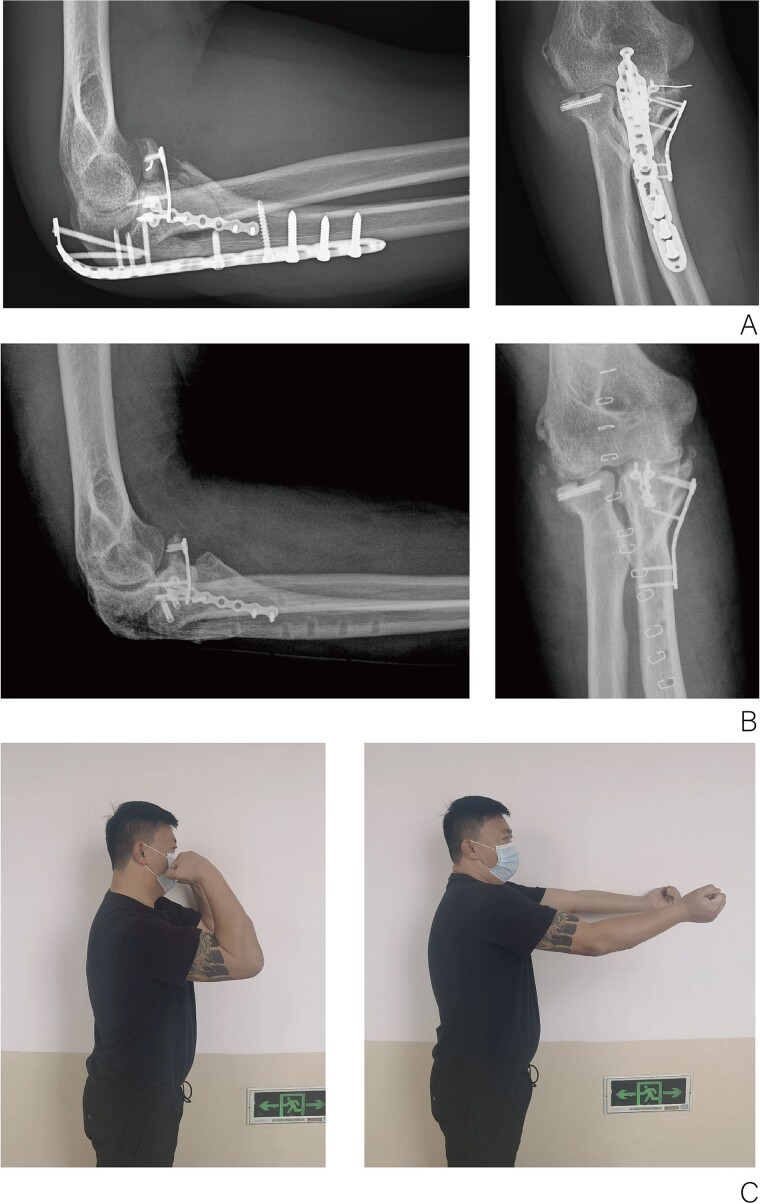
(A) X-ray of elbow at 3 months after surgery. (B) X-ray of elbow at 1 year after surgery. (C) Function of elbow at 6 months after surgery.

Passive range-of-motion exercises for the elbow were initiated 1–2 days postoperatively, with progressive elbow strength training commencing 6–8 weeks following the removal of the brace. A follow-up X-ray examination conducted three months postoperatively demonstrated the absence of bone resorption at the coronoid process fracture site, with all fracture components securely fixed. Furthermore, no complications, including Reflex Sympathetic Dystrophy or ulnar nerve paralysis, were observed (Fig. 2A). At 1 year postoperatively, partial internal fixation was removed, and follow-up X-ray imaging revealed successful bony union of the coronoid process fragment, with no notable bone resorption (Fig. 2B). The elbow joint demonstrated a range of motion, flexing to 120° and extending to −15° (Fig. 2C).

## Discussion

In this case, the patient, who was overweight, sustained trauma directly to the proximal ulna near the joint, resulting in a high-energy injury localized around the joint. The patient’s elbow, positioned in slight flexion, was subjected to high-energy force, which led to a comminuted fracture of the proximal ulna and posterior dislocation of the elbow joint. This led to impingement of the coronoid process and the distal articular surface of the humerus, resulting in a comminuted fracture of the coronoid process. Additionally, the anterior aspect of the radial head impinged upon the trochlea, causing a fracture of the radial head. Nevertheless, no excessive varus, valgus, or rotational forces were applied to the medial and lateral collateral ligaments of the elbow throughout the procedure, thus preventing severe ligament damage. Prior to the surgery, extensive discussions were conducted with the patient, during which the surgical plan was thoroughly explained. The patient’s informed consent was obtained through the signing of a consent form.

The choice of internal fixation techniques should be guided by considerations including the dimensions of the bone graft, the morphology of the fracture, the quality of the bone tissue, and the specific characteristics of the injury. In the absence of comprehensive biomechanical studies, hollow screw fixation continues to be a prevalent method for the reconstruction of the coronoid process [2]. In certain instances, reconstruction may necessitate the use of alloplastic or autogenous iliac bone, or alternatively, a prosthetic replacement of the coronoid process may be employed [3–5].

**Figure 3:**
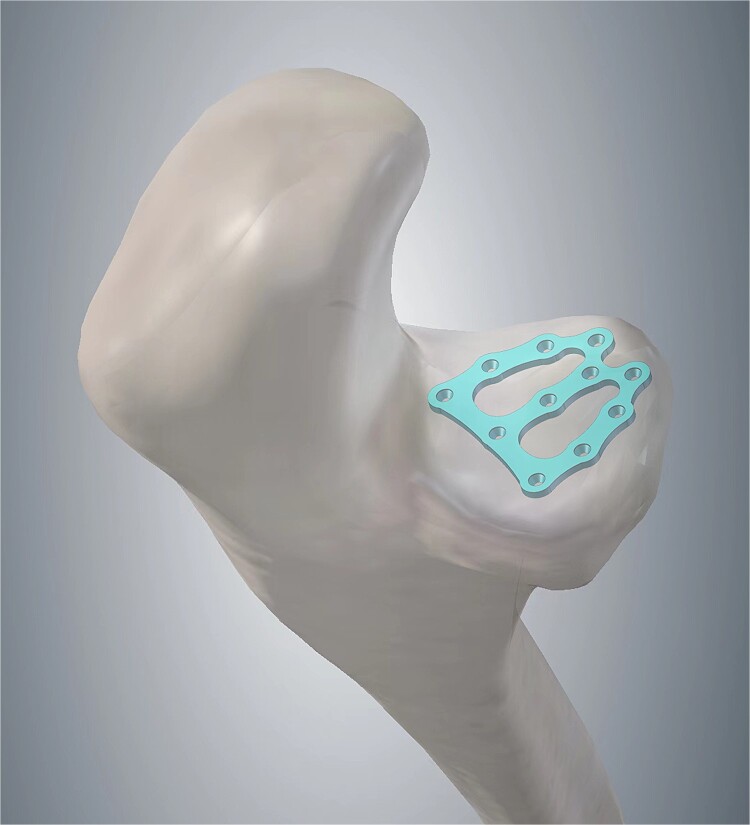
Patent drawing of the mini-plate for intra-osteal fixation of comminuted coronoid process fracture.

In this case, a posterior approach was utilized, facilitating the treatment of the radial head fracture and the ulnar component of the lateral collateral ligament through lateral stripping, while addressing the coronoid process fracture medially. However, the coronoid process in this patient had fragmented from its base into three distinct blocks, each measuring 10 × 10 mm^2^. Each fractured segment of the coronoid process plays a crucial role in maintaining anterior stability of the elbow. The proximal ulna exhibited fractures beyond the olecranon, and there were no suitable stress-bearing surfaces available for suture anchors to simultaneously secure all coronoid fragments. Only plate fixation provides simultaneous reinforcement of the articular surface in both horizontal and sagittal planes, thereby enhancing surface stability and facilitating early functional exercises for the patient. Consequently, we employed intra-osteal fixation with a mini plate to stabilize the coronoid fracture blocks in conjunction with the olecranon, reconstructed the articular surfaces of both the olecranon and coronoid processes, and utilized a mini plate and locking compression plate to secure the remaining fragment, achieving substantial restoration of the bony architecture of the proximal ulna. This technique not only minimizes operative time but also reduces soft tissue damage, which has significant implications for future fracture healing and rehabilitation processes. Nevertheless, no studies to date have investigated the application of osteosynthesis plate intra-osteal fixation, and the adherence of this fixation method to established ethical treatment principles remains to be determined. At present, the patient’s elbow joint fracture has fully healed without complications, and the elbow joint has demonstrated favorable functional recovery. Based on this surgical approach, we have developed an internal fixation osteosynthesis plate specifically for treating comminuted coronoid process fractures and have filed a patent application (Fig. 3).

In conclusion, we propose that for intricate elbow fractures involving a comminuted coronoid process, mini plate intra-osteal fixation represents a viable alternative that allows for the potential completion of the surgical intervention through a single approach, addressing both the coronoid process and the proximal ulna.
